# The Role of Insulin-Like Growth Factor 1 in the Progression of Age-Related Hearing Loss

**DOI:** 10.3389/fnagi.2017.00411

**Published:** 2017-12-12

**Authors:** Lourdes Rodríguez-de la Rosa, Luis Lassaletta, Miryam Calvino, Silvia Murillo-Cuesta, Isabel Varela-Nieto

**Affiliations:** ^1^“Alberto Sols” Biomedical Research Institute CSIC-UAM, Madrid, Spain; ^2^Centro de Investigación Biomédica en Red de Enfermedades Raras, Instituto de Salud Carlos III, Madrid, Spain; ^3^Hospital La Paz Institute for Health Research (IdiPAZ), Madrid, Spain; ^4^Otorhinolaryngology Department, Hospital La Paz, Madrid, Spain

**Keywords:** ARHL, GH, IGF system, presbycusis, rare diseases

## Abstract

Aging is associated with impairment of sensorial functions and with the onset of neurodegenerative diseases. As *pari passu* circulating insulin-like growth factor 1 (IGF-1) bioavailability progressively decreases, we see a direct correlation with sensory impairment and cognitive performance in older humans. Age-related sensory loss is typically caused by the irreversible death of highly differentiated neurons and sensory receptor cells. Among sensory deficits, age-related hearing loss (ARHL), also named presbycusis, affects one third of the population over 65 years of age and is a major factor in the progression of cognitive problems in the elderly. The genetic and molecular bases of ARHL are largely unknown and only a few genes related to susceptibility to oxidative stress, excitotoxicity, and cell death have been identified. IGF-1 is known to be a neuroprotective agent that maintains cellular metabolism, activates growth, proliferation and differentiation, and limits cell death. Inborn IGF-1 deficiency leads to profound sensorineural hearing loss both in humans and mice. IGF-1 haploinsufficiency has also been shown to correlate with ARHL. There is not much information available on the effect of IGF-1 deficiency on other human sensory systems, but experimental models show a long-term impact on the retina. A secondary action of IGF-1 is the control of oxidative stress and inflammation, thus helping to resolve damage situations, acute or made chronic by aging. Here we will review the primary actions of IGF-1 in the auditory system and the underlying molecular mechanisms.

## IGF system, upstream regulation, and downstream IGF-1 signaling

The mammalian IGF system is comprised of insulin-like growth factors (IGF), receptors and binding proteins (IGFBP). IGFs and insulin are small polypeptides produced as pre-pro-peptides that can bind the insulin (IR) and IGF-1 (IGF1R) tyrosine kinase receptors. IGFs also bind the cation-independent mannose-6-phosphatase IGF-2 receptor (IGF2R; Foulstone et al., 2005). The biological actions of IGFs are primarily mediated by binding to the IGF1R, a heterotetramer with extracellular IGF binding domains and intracellular tyrosine kinase domains. The carboxy-terminal domain has docking sites for intracellular substrates (IRS1-4/SHC; Laviola et al., 2007) that, in turn, bind and activate a network of intracellular signaling molecules. Factor-receptor interactions are modulated by binding proteins (IGFBPs) and associated proteases. In plasma, IGFBPs carry IGFs and these regulate their half-life, distribution and biological actions. IGFBPs control the bioavailability of IGF-1 to its receptors by competing with receptors for free factors (Firth and Baxter, 2002).

The growth hormone (GH) is a peptide hormone secreted by somatotroph cells of the pituitary gland. GH stimulates the growth of all body tissues, thus its level rises progressively during childhood and peaks at puberty. IGF-1 is secreted by the liver as a result of stimulation by GH, which binds to its receptor, GHR, inducing homodimerization and initiating signal transduction through receptor-associated Janus kinase (JAK) 2 (Herrington et al., 2000). Activation of signal transducers and transcription activators of STAT5b family members is critical for regulating liver IGF-1 gene expression (Davey et al., 2001), as well as for the transcriptional regulation of other IGF system genes, including those coding for IGFBP3 and IGFALS. ALS is a secreted hepatic protein that can be found freely circulating or forming a ternary complex with IGF-1 and IGFBP-3, thus prolonging the half-life of IGF-1 and decreasing its availability to tissues (Boisclair et al., 2001).

At the cell surface, IGF-1 binds to high affinity IGF1R, inducing autophosphorylation and allowing docking of the receptor substrates IRS-1 to IRS-4, Grb2-associated binder 1 (Gab-1) and the Src homology 2 domain containing protein (SHC). Activated docking proteins subsequently recruit cytoplasmic components of downstream signaling pathways, including the MAPK (RAF-MEK-ERK1/2 and p38) and the PI3K-AKT pathways, and transduce the IGF signaling (Siddle, 2011). Depending on the cellular context, IGF-1 regulates different processes. For example, IGF-1 modulates gene expression in chondrocytes (Yang et al., 2017), protein synthesis in osteoblasts (Guo et al., 2017), cell cycle in enterocytes (Van Landeghem et al., 2015), metabolism in adipocytes (Chang et al., 2016), survival in cochlear hair cells (Yamahara et al., 2017) and autophagy in otic neural precusors (Aburto et al., 2012b). IGF1R can also translocate to the nucleus, activate transcription and regulate gene expression (Sehat et al., 2010).

Analysis of downstream signaling in the deaf *Igf1*^−/−^ mouse has further contributed toward understanding its cochlear actions and demonstrated that an IGF-1 deficit is associated with activation of the p38 MAPK pathway (related to the cellular response to stress). RAF-ERK1/2 and AKT activity (cell proliferation and survival) are also impaired (Sanchez-Calderon et al., 2010) but the overall autophagic flux is unaffected (de Iriarte Rodríguez et al., 2015b; Magariños et al., 2017). Further analysis of IGF-1 signaling has been carried out by studying null mice for downstream targets such as IRS1 (Tang et al., 2017), IRS2, PTP1B (Murillo-Cuesta et al., 2012), GRF1 (Fernández-Medarde et al., 2009), and CRAF (De Iriarte Rodriguez et al., 2015a). Cochlear comparative transcriptomics have unveiled the role of FoxM1 and FoxP3 forkhead box transcription factors, and myocyte enhancer factor-2 (MEF-2) in inner ear development as well as IGF-1 cochlear actions (Sanchez-Calderon et al., 2010). The impact of IGF-1 deficiency depends on the tissue and organs studied. In the *Igf1*^−/−^ mouse bone, cell survival and AKT signaling are gravely affected, but RAF kinase-mediated proliferation is not (Rodríguez-de La Rosa et al., 2014). In the mouse retina, however, IGF-1 deficit causes impairment of the autophagic flux, leading to increased inflammation, apoptosis and age-associated blindness (Rodriguez-de La Rosa et al., 2012; Arroba et al., 2016). Finally, analysis of the *Igf1*^−/−^ vestibule has confirmed the role of p38 and added new players such as p53 and microRNAs (Rodríguez-de la Rosa et al., 2015).

In summary, IGF-1 has a role in brain development and maturation (Dyer et al., 2016). Later in life, bioactive IGF-1 circulating levels are reduced, a trend that has been associated with human frailty and cognitive decline (Vestergaard et al., 2014). IGF-1 deficiency leads to increased inflammation and to the failure of intracellular cell renewal mechanisms. This is critical in the inner ear because, as discussed below, none of the main cell types essential for hearing regenerate (Mittal et al., 2017).

## Inner ear development, adult anatomy, and age-associated degeneration

The inner ear develops from the otic ectodermal placode that invaginates to form the otic vesicle. This transitory embryonic structure contains the information required to generate most cell types of the adult inner ear, including the sensory cells and the auditory-vestibular neurons (Kelly and Chen, 2009; Magariños et al., 2012, 2014; Burns et al., 2015). Development of the inner ear is tightly regulated by intrinsic and extrinsic factors (Sanchez-Calderon et al., 2007; Gálvez et al., 2017). Among these factors, IGF-1 promotes proliferation and survival of otic progenitor cells, supports neurogenesis and facilitates late differentiation in species from fish to humans (Ayaso et al., 2002; Schlueter et al., 2007; Zou et al., 2009; Aburto et al., 2012a; Varela-Nieto et al., 2013; Tafra et al., 2014). IGF-1 plays a key role in brain development and maintenance of stem cells (Nieto-Estévez et al., 2016).

The adult inner ear comprises the cochlea and the vestibular organ, which are responsible for hearing and equilibrium, respectively. The organ of Corti in the cochlea contains highly specialized hair cells (inner -IHC- and outer -OHC-), which transform mechanical sounds into electrochemical signals that are conveyed to the brain by the VII vestibulocochlear cranial nerve (Stephenson, 2012). Sound induces the movement of hair cell stereocilia, causing the opening of ion channels, an influx of K^+^ ions and depolarization. Depolarization of IHC results in glutamate release and synapse with 10–30 afferent auditory bipolar neurons. Meanwhile, OHCs amplify the incoming sound stimulation and enhance frequency selectivity of the cochlear response (Fettiplace and Kim, 2014; Reichenbach and Hudspeth, 2014). The organ of Corti is connected to the brain by two types of neurons in the spiral ganglion (SGN; Coate and Kelley, 2013). Type I and type II neurons innervate IHC and OHC respectively. Of these two kinds of neurons, type I is the most abundant (95%). The SGN axons form the cochlear branch of the vestibulocochlear nerve and connect the peripheral spiral ganglia with the cochlear nuclei at the brainstem. Sound information progresses in a complex multisynaptic, parallel, and ascendant pathway from the cochlea through the brainstem nuclei to the auditory cortex, preserving the tonotopic organization (Tsukano et al., 2017).

Expression levels of IGF-1 and its high affinity receptor, IGF1R, are elevated during late cochlear development, decline significantly after birth, but baseline expression is maintained throughout the organism's life (Murillo-Cuesta et al., 2011; Okano et al., 2011). In the mouse cochlea, IGF-1 is clearly detected in spiral ganglion neurons and stria vascularis, and its expression is modulated with aging (Riva et al., 2007). IGF binding proteins are also expressed in the developing ear and throughout life (Okano and Kelley, 2013). Finally, IGF system elements are expressed in the vestibular system over a similar time course (Degerman et al., 2013; Rodríguez-de la Rosa et al., 2015).

During aging, peripheral and central auditory structures degenerate, leading to ARHL (Fetoni et al., 2011). The primary pathological alterations observed in mouse models include progressive degeneration and loss of HC and SGN (Bao and Ohlemiller, 2010; Bowl and Dawson, 2015), as well as changes in the central auditory pathway. Typically, presbycusis debuts with loss of OHC, mainly in basal cochlear regions (high frequencies), and extends toward the apex and IHC (Ohlemiller and Gagnon, 2004). OHC defects result in a moderate increase of the hearing threshold, whereas defects in IHC or the auditory neurons can lead to profound deafness (Ouda et al., 2015). Swelling of the afferent nerve terminals and a decrease in the density of their associated ribbon synapses causes a synaptopathy that is sometimes the primary defect (Wan and Corfas, 2015).

The stria vascularis is a three-cell layer structure within the cochlea that maintains the K+ concentration and the endocochlear potential (Magariños et al., 2012). During aging, the stria vascularis shows disorganization and atrophy, with loss of marginal cells and progressive merging of strial capillaries (Ohlemiller, 2009). In addition, thinning, degeneration of fibrocytes and loss of capillaries are observed in the spiral ligament (Hequembourg and Liberman, 2001). In human cohorts, decreased age-related-IGF-1 bioavailability correlates with progression of hearing impairment (Lassale et al., 2017), as will be discussed in depth below. Studies carried out with *Igf1*^−/−^ and *Igf1*^−/+^ mice confirmed this trend and showed acceleration in the damage of the neural structures and stria vascularis (Riquelme et al., 2010).

The molecular mechanisms underlying ARHL that have been described include redox imbalance, accumulation of mitochondrial DNA damage, and excitotoxicity, leading to apoptotic and necrotic cell death (Menardo et al., 2012; Wong and Ryan, 2015). Interestingly, experimental models indicate that anti-oxidant therapy and control of micronutrients could prevent or ameliorate ARHL (Fetoni et al., 2009; Guastini et al., 2011; Ding et al., 2016). These mechanisms are similar to those involved in drug- and noise-induced hearing loss (Frisina et al., 2016; Kalinec et al., 2017).

The central auditory system shows alterations that could be secondary to the diminished input from the damaged periphery. These include modifications in the expression of calcium binding proteins (parvalbumin, calbindin and calretinin, and glutamate-decarboxylase), atrophy of the gray and white matter and changes in the content of some metabolites (Ouda et al., 2015). However, a minimal age-related decline in the total number of neurons in central auditory structures has been reported. IGF-1 expression is modulated by cochlear damage in the auditory central pathway (Alvarado et al., 2007) and its deficit also impacts neurotransmission in the cochlear nucleus (Fuentes-Santamaria et al., 2013, 2016).

To summarize, IGF system elements are expressed in the inner ear and central auditory pathway, and their expression is regulated by damage and age in different species. The importance of this system in hearing loss has been further proven by the study of human mutations and human cohorts as described below.

## Association of the IGF system with human genetic deafness and age-related hearing loss

ARHL is a multifactorial process that results in cochlear damage over the span of a life. Noise, ototoxic agents, trauma, vascular insults, metabolic changes, hormones, diet, immune system, and genetic predisposition are all contributing factors (Gates and Mills, 2005; Fetoni et al., 2011). Given the increase in the average age of the population as well as in noxious environmental agents, the impact of ARHL is continuously growing.

The relationship between nutrition and ARHL is an emerging interdisciplinary field and recent evidence points to vitamin imbalances and high fat diets as risk factors (Partearroyo et al., 2017). In addition, the genetic study of ARHL is an expanding field that has rendered several gene candidates thus far (Salminen and Kaarniranta, 2010; Op De Beeck et al., 2011; Fransen et al., 2015; Koffler et al., 2015).

Mutations in human genes coding for IGF-1, IGF1R, and other members of the GH/IGF-1 system cause rare diseases (Tables 1, 2), which normally have an early onset. Patients show growth retardation and frequent microcephaly. Interestingly, only when IGF-1 actions are totally impaired do the affected patients show syndromic hearing loss (Table 1). Indeed, of the 7 mutations described in young humans, hearing phenotype of 2 patients have not been reported (Van Duyvenvoorde et al., 2010, 2011; Shaheen et al., 2014) and 3 show low to normal IGF-1 levels, which show reduced affinity for IGF1R binding in the case tested, and normal hearing (Netchine et al., 2009; Fuqua et al., 2012; Batey et al., 2014). No data have been published on the evolution of hearing loss associated with aging that these patients present. Finally, 2 patients showed severe hearing loss which was associated in one case with total absence of circulating IGF-1 (Woods et al., 1996) and in the other with an extremely low binding affinity (Walenkamp et al., 2005). Accordingly, haploinsufficient IGF-1 availability causes growth retardation and has been associated with short adult stature and hearing loss in other genetic syndromes such as Noonan's or Turner' syndromes (Barrenäs et al., 2000, 2005b; Welch and Dawes, 2007; El Bouchikhi et al., 2016).

**Table 1:** Reported mutations of the *IGF1* gene.

	**(Woods et al., 1996)**	**(Batey et al., 2014)**	**(Walenkamp et al., 2005)**	**(Netchine et al., 2009)**	**(Shaheen et al., 2014)**	**(Fuqua et al., 2012)**	**(Van Duyvenvoorde et al., 2010, 2011)**
Mutation type	Homozygous Deletion	Heterozygous Deletion	Homozygous Missense mutation	Homozygous Missense mutation	Homozygous Missense mutation	Heterozygous Splicing mutation	Heterozygous Insertion
Mutational analysis	181-bp deletion of *IGF1* gene (ex 4–5)	262-kb deletion of chr 12 (*IGF1* gene)	Val44Met c.274G?A, p.V44M	Arg36Gln c.251G?A, p.R36Q (ex 4)	Arg98Trp c.292C?T, p.R98W	Splicing excision ex 4 c.402+1G?C, p.N74Rfs^*^8	Stop codon ex 3 c.243-246dupCAGC, p.S83Qfs^*^13
Clinical data	♂ 15.8 yr Intrauterine growth restriction Postnatal growth failure Microcephaly Micrognathia Clinodactyly Mild myopia Cognitive delay	♂ 2.3–8.4 yr Intrauterine growth restriction Postnatal growth failure Microcephaly Micrognathia Clinodactyly Cognitive delay	♂ 55 yr Intrauterine growth restriction Progressive postnatal growth failure Microcephaly Dysmorphic features Severe cognitive delay Deaf-mutism	♂ 11 mo-9 yr Intrauterine growth restriction Progressive postnatal growth failure Microcephaly Non-dysmorphic features Clinodactyly Mild cognitive delay	♀ 2.8 yr (sibling-1) ♂ 5 yr (sibling-2) Primordial dwarfism (severe prenatal and postnatal growth deficiency)	♂ 8.8 yr Severe postnatal growth failure Normal physical examination Normal cognitive development	♀ 8.2 yr (sibling-1) Slow motor development Poor growth Delayed bone age ♂ 6.2 yr (sibling-2) Poor growth Delayed bone maturation
Consanguinity	Yes	No	Yes	Yes	Yes	No	No
Birth weight (kg)	1.4 (−3.9SD)	2.7 (−1.5SDS)	1.4 (−3.9SDS)	2.3 (−2.4SDS)	Sib-1: 1.6 (−3.5SD) Sib-2: ND	3.0 (−1.5SDS)	Sib-1: 2.3 (−2.9SDS) Sib-2: 3.3 (-1.2SDS)
Birth length (cm)	37.8 (−5.4SD)	47.6 (−1.2SDS)	39 (-4.3SDS)	44 (−3.7SDS)	ND	47 (-0.6SDS)	Sib-1: 44 (−3.8SDS) Sib-2: 50 (−1.0SDS)
Growth weight (kg)	15.8 yr: 23 (−6.5SD)	2.3 yr: 8.8 (−3.8SDS) 5.3 yr: 14.4 (−2.5SDS) 8.4 yr: 21.9 (−1.5SDS)	ND	11 mo: 5.3 (−5.0SDS) 2.8 yr: 7.0 (−7.0SDS)	2.8 yr, sib-1: 8.2 (−4.1SD) 5 yr, sib-2: 9 (−4.9SD)	8.8 yr: 21 (−2.1 SDS)	ND
Growth height (cm)	15.8 yr: 119.1 (−6.9SD)	2.3 yr: 77.5 (−3.1SDS) 5.3 yr: 96.9 (−2.9SDS) 8.4 yr: 114.9 (−2.7SDS)	55 yr: 117.8 (−8.5 SDS)	11 mo: 64 (−3.7SDS) 2.8 yr: 76 (−4.9SDS)	2.8 yr, sib-1: 81 (−3.2SD) 5 yr, sib-2: 89 (−4.3SD)	8.8 yr: 109 (−4.0 SDS)	8.2 yr, sib-1: 108.9 (−4.1 SDS) 6.2 yr, sib-2: 98.7 (−4.6 SDS)
Hearing impairment	15.8 yr: severe bilateral HL	Normal hearing	55 yr: severe bilateral HL	9 yr: normal hearing	ND	Normal hearing	ND
IGF-1 levels (ng/mL)	Undetectable 15 yr: ?3	Low-normal 2.3 yr: 43.7; 5.3 yr: 58.5 8.4 yr: 100	Very high 55 yr: 606 (+7.3SDS)	Low 2.7 yr: 11 (before GH treatment)	ND	Low-normal 9.3 yr: 115 (−2.2SDS) (before GH treatment)	Low 8.2 yr, sib-1: 76 (−2.3SDS) 6.2 yr, sib-2: 35 (−2.6SDS)
IGFBP-3 levels (mg/L)	Normal 15 yr: 3.3	Normal 5.3 yr: 4.3 8.4 yr: 5.4	Normal 55 yr: 1.98 (+0.1SDS)	Normal to high (after GH treatment)	ND	Normal 9.3 yr: 2.4 (−1.2SDS) (before GH treatment)	Normal 8.2 yr, sib-1: 3.6 (1.2SDS) 6.2 yr, sib-2: 2.1 (0.1SDS)
ALS levels (mg/L)	Normal	Normal 5.3 yr: 10	High 55 yr: 28.9 (+3.4SDS)	Normal to high (after GH treatment)	ND	Normal to high 9.3 yr: 13 (before GH treatment)	Normal 8.2 yr, sib-1: 20.1 (1.1SDS) 6.2 yr, sib-2: 11.5 (-0.4SDS)
IGF-1 affinity for IGF1R	ND	ND	Extremely low 90-fold lower	Partially reduced 3.9-fold lower	ND	ND	No affinity

*Overview of homozygous and heterozygous mutations described in the human IGF1 gene. HL, hearing loss; mo, month; ND, not determined; SD, standard deviation; SDS, standard deviation score; sib, sibling; yr, year. Hearing loss was observed in patients with undetectable or very high IGF-1 levels (2 out 7 cases)*.

**Table 2:** GH/IGF-1 axis and deafness.

**Gene**	**HGDM^®^ mutations (number)**	**General phenotype**	**Deafness mutation**	**Clinical cases**	**Auditory phenotype**	**References**
*GHRHR*	Missense (26) Splicing (10) Regulatory (4) Small del (4) Small ins (2) Gross del (1)	Severe dwarfism Central obesity ↑ LDL cholesterol levels ↑ Systolic blood pressure ↓Cranial volume, frontal bossing ↓Depth of skull, ↓facial height, Laryngopharyngeal reflux Laryngeal constriction ↓↓ GH and IGF-1 serum levels	Homozygous splice mutation (c.5711 G.A)	26 (13 ♂, 13 ♀) 47.6 ± 15.1 yr	↑ prevalence of dizziness Early mild high-tone SNHL ↓Stapedius reflex ↓ Transient evoked otoacoustic emissions	Prado-Barreto et al., 2014
*GHR*	Missense (56) Splicing (21) Small del (4) Small ins (4) Gross del (9) Complex (1)	Laron syndrome Dwarfism Obesity Hypogenitalism Hypoglycemia at birth and in early infancy	GH-R-W-15X (exon 2) GH-R-R217X (exon 7) GH-R-3,5,6 exon del GH-R-43X/Norm (exon 4)	5 untreated (2 ♂, 3 ♀), 49.2 ± 4.8 yr 1 with delayed rhIGF-1 treatment (♂, 15 yr)	Hearing impairment (50% low-tone SNHL; 16.6% high-tone SNHL; 25% combined high-/low-tone SNHL; 8.3% mixed HL)	Attias et al., 2012
*PTPN11*	Missense (111) Splicing (4) Small del (7) Small ins (1) Small indels (3) Gross del (1) Gross ins (3) Complex (2) Repeats (1)	Noonan syndrome (craniofacial dysmorphic features, short stature, congenital heart defects, including pulmonary stenosis) Leopard syndrome lentigines; Electrocardiogram conduction abnormalities; ocular hypertelorism; pulmonary stenosis; abnormalities of the genitalia; retardation of growth SNHL (15–25% of patients with Leopard syndrome)	Heterozygous missense mutation (c.1381G>A)	5 yr ♀	Bilateral profound SNHL Enlarged vestibular aqueducts	Chu et al., 2013
			Missense mutation (c.836A>G, Tyr279Cys)	17 yr ♂	Severe bilateral SNHL	Martínez-Quintana and Rodríguez-González, 2012
			Missense mutation in exon 7 (836A3G; Tyr279Cys)	16 yr ♂	Severe bilateral SNHL	Kim et al., 2011
			Mutation c.1510A > G Mutation c. 124A > G Mutation c. 922A > G Mutation c. 124A > G Mutation c. 836A > G	Neonatal ♂ Neonatal ♂ Neonatal ♀ 1 yr ♀ Neonatal ♀	Severe congenital HL Severe congenital HL Severe congenital HL and multiple ear infections. Progressive SNHL, otitis media Severe congenital HL	Van Nierop et al., 2017
			c.124A>G c.179 G>C, c.181G>A, c.182A>G c.186A>G, c.236A>G c.417G>C c.794G>A, c.922A>G, c.923A>G c.1504T>A, c.1510A>G	Both sexes Variable age	Temporary hearing impairment (sensorineural, Conductive, mixed) External ear anomalies (81%)	van Trier et al., 2015
*IGF1R*	Missense (36) Splicing (1) Regulatory (1) Small del (1) Small ins (4) Gross del (12) Gross ins (5) Complex (2)	Moderate- severe growth failure Mild intellectual impairment Microcephaly Dysmorphic features Cardiac malformations Disturbed glucose tolerance Failure to thrive	Deletion p.A711_E714del	Two siblings, 11 yr ♀and 7 yr ♂	Deafness	Maystadt et al., 2015
			Complete deletion (15q26.3, exons 1–21) Partial deletion (exons 3–21)	3 yr ♀ 2 yr ♂	Bilateral HL Recurrent ear infections	Ester et al., 2009

*Reported cases of hearing impairment in patients with mutations in members of the GH/IGF-1 axis, except for IGF-1. Del, deletion; HL, hearing loss; Ins, insertion; SNHL, sensorineural hearing loss; yr, year*.

Human mutations in the gene coding for the high affinity receptor IGF1R are even less frequent and normally found in heterozygosis (Table 2; Abuzzahab et al., 2003; Ester et al., 2009; Klammt et al., 2011; Fang et al., 2012; Gannagé-Yared et al., 2013; Prontera et al., 2015). Reduced bioactive IGF-1 levels caused by mutations in related genes, such as those coding for GH and the GHR, are also associated with hearing loss (Table 1; Giordano et al., 2015; Muus et al., 2017). In general, the authors have not done a thorough study of sensory functions, including hearing. However, it is worth noting that Laron's syndrome patients have been studied in depth and show early onset of ARHL (Attias et al., 2012). Laron's syndrome is an autosomal recessive human disorder characterized by mutations in the GHR that cause insensitivity to GH stimuli and, in turn, extremely low IGF-1 synthesis in the liver. Patients have a short stature, among other characteristics, and normal hearing at young ages but develop early onset ARHL, which could be prevented by IGF-1 treatment. Still, much work is needed to fully understand the relationship between genes associated with short stature (Baron et al., 2015; Wit et al., 2016) and hearing loss.

Finally, epidemiologic studies of aging cohorts have shown a relationship between bioactive IGF-1 and hearing loss (Lassale et al., 2017). Furthermore, there is increasing evidence linking these two factors with cognitive decline and onset of neurodegenerative diseases (Dik et al., 2003; Watanabe et al., 2005; Fortunato et al., 2016; Lassale et al., 2017; Wrigley et al., 2017), dementia (Peracino, 2014; Su et al., 2017), depression (Van Varsseveld et al., 2015; Kim et al., 2017), short stature (Barrenäs et al., 2005a; Crowe et al., 2011), cardiovascular pathologies (Burgers et al., 2011; Tan et al., 2017), and aging (Bainbridge and Wallhagen, 2014; Vestergaard et al., 2014).

These data lead us to pose the following questions: (i) which cochlear processes are IGF-1-dependent and dramatically impaired during aging? And (ii) is there a potential for prevention and repair interventions based on IGF-1 targets? Insight into the first question has been obtained from the study of the *Igf1*^−/−^ mouse that shows profound syndromic bilateral sensorineural hearing loss (Cediel et al., 2006). The main cellular alterations reported were delayed postnatal development, a significant decrease in the number and size of auditory neurons, aberrant innervation patterns, increased neural apoptosis, deficits in myelination and increased efficacy of glutamatergic synapses (Camarero et al., 2001, 2002; Sanchez-Calderon et al., 2007; Fuentes-Santamaria et al., 2016). *Igf1*^−/−^ mice develop further cellular degeneration with aging. As bioactive IGF-1 levels decrease, the heterozygous mice also show an accelerated hearing loss secondary to degeneration of the SGN (Riquelme et al., 2010). Our findings support the idea that IGF-1 levels may hold predictive value for the stratification of ARHL and, secondary to hearing loss, for cognitive decline. Regarding the aforementioned mechanisms, IGF-1 is a neurotrophic factor with anti-inflammatory actions. It is anti-apoptotic and favors cell renewal.

In this context, IGF-1 emerges as a potential protector of the inner ear. Studies in animal models have shown that local application of recombinant human IGF-1 (rhIGF-I) protects the cochlea from functional and histologic losses induced by aminoglycoside ototoxicity and noise exposure (Iwai et al., 2006; Yamahara et al., 2015). IGF-1 rescued hair cells from apoptosis by downregulating pro-apoptotic gene expression and regulating glucose transporters (Yamahara et al., 2015). Similarly, IGF-1 and substance P protect vestibular hair cells against neomycin ototoxicity (Yoshida et al., 2015). Recombinant IGF-1 therapy has been approved to increase linear growth, and therefore height, in humans, spurring an increasing interest in the potential use of IGF-1 for the treatment of hearing loss (Yamahara et al., 2015). IGF-1 potential has been studied in patients with sudden sensorineural hearing loss who were resistant to treatment with systemic glucocorticoids (Nakagawa et al., 2012, 2014). A recent study with 120 patients concluded that treatment with topical IGF-1 therapy had significant effects on hearing recovery depending on their age (< 60 years) and early initiation of salvage treatment (Nakagawa et al., 2016). Prolonged activation of IGF1R by treatment with IGF-1 or analogs might have undesired secondary effects, thus their potential use to delay aging maybe limited. However available data highlight the interest of exploring IGF-1 downstream targets as drug candidates for ARHL.

## Author contributions

LR-dlR, LL, MC, SM-C, IV-N: drafted the work and wrote the manuscript; LR-dlR, SM-C, and IV-N: revised the manuscript. All authors approved the manuscript in its final form.

### Conflict of interest statement

The authors declare that the research was conducted in the absence of any commercial or financial relationships that could be construed as a potential conflict of interest.
